# Association between serum 25-hydroxyvitamin D and osteoarthritis: A national population-based analysis of NHANES 2001–2018

**DOI:** 10.3389/fnut.2023.1016809

**Published:** 2023-02-28

**Authors:** Guoyu Yu, Yuan Lin, Hanhao Dai, Jie Xu, Jun Liu

**Affiliations:** ^1^Clinical College of Orthopedics, Tianjin Medical University, Tianjin, China; ^2^Department of Orthopedics, Fujian Provincial Hospital, Shengli Clinical Medical College, Fujian Medical University, Fuzhou, China; ^3^Department of Joints, Tianjin Hospital, Tianjin University, Tianjin, China

**Keywords:** 25(OH)D, NHANES, vitamin D, supplement, osteoarthritis, modification, obesity

## Abstract

**Background:**

Previous studies have not provided a consensus on the effect of serum 25-hydroxyvitamin D [25(OH)D] on osteoarthritis (OA). We aimed to evaluate the association using a large, nationally representative sample.

**Methods:**

The cross-sectional data were obtained from the 2001 to 2018 National Health and Nutrition Examination Survey (NHANES). Individuals aged ≥40 years who had information of serum 25(OH)D, self-report OA, and related covariates were included. Multivariable logistic regression analysis was employed to assess the association between serum 25(OH)D and osteoarthritis.

**Results:**

Among the 21,334 participants included (weighted mean age, 56.9 years; 48.5% men), the proportion of participants with high serum 25(OH)D concentrations (≥100 nmol/L) increased significantly from 4.2% in 2001–2006 to 18.8% in 2013–2018. Higher serum 25(OH)D levels were associated with more osteoarthritis prevalence in fully adjusted model (odd ratio [OR] 1.25 [95% CI: 1.10, 1.43] for the 50–75 nmol/L group; OR 1.62 [95% CI: 1.42, 1.85] for the 75–100 nmol/L group; OR 1.91 [95% CI: 1.59, 2.30] for the ≥100 nmol/L group; with <50 nmol/L group as the reference) (*p* < 0.001 for trend). The association was consistent across several sensitivity analyses, including propensity score methods and excluding participants who had received vitamin D supplement. In subgroup analysis, the OR for the association increased significantly with body mass index (BMI) (BMI < 25 kg/m^2^, 1.01 [95% CI: 1.04, 1.08]; BMI 25–30 kg/m^2^, 1.05 [95% CI: 1.01, 1.08]; BMI ≥ 30 kg/m^2^, 1.10 [95% CI: 1.06, 1.13]; *p* = 0.004 for interaction).

**Conclusion:**

There was a positive correlation between serum 25(OH)D and osteoarthritis with a possible modification by BMI. Our finding raises concerns about the potential adverse effects of high serum 25(OH)D on osteoarthritis, particularly among obese individuals. More well-designed studies are still needed to validate our findings in future.

## 1. Introduction

More than 32.5 million US adults suffer from osteoarthritis (OA), which places a heavy burden on society and the economy (1). OA risk factors can be divided into person-level factors (age, gender, genetics, and obesity) and joint-level factors (injury, malalignment, and abnormal loading), which interact with each other in a complex way (2). Although the underlying causes of OA are still unclear, the disease is regarded as “wear and tear” arthritis characterized by a gradual loss of cartilage, inflammation of the synovium, osteophyte formation, and subchondral bone changes (3). There are currently no disease-modifying medications available in clinical practice, emphasizing the necessity of identifying risk factors associated with OA for disease prevention and treatment.

As a steroidal hormone, vitamin D has diverse biological effects on a variety of tissues (4). Its primary function is thought to regulate bone metabolism and calcium homeostasis. Thus, vitamin D abnormalities probably impede subchondral bone growth and remodeling, which play a critical role in the pathogenesis of OA (3). Vitamin D is also believed to affect inflammation and muscle strength (4), which are involved in OA progression. Vitamin D has received extensive attention for its role in osteoarthritis pathogenesis, since it has such a potential impact on bone, inflammation, and muscle (5).

Previous researches have yet to achieve consensus on the effect of serum 25-hydroxyvitamin D [25(OH)D] on the incidence or development of OA. Some cross-sectional and longitudinal studies have shown that vitamin D deficiency is associated with osteoarthritis (6–8), yet others did not (9, 10). Additionally, three large, long-term cohort studies have shown that individuals with higher serum 25(OH)D have an increased risk of OA or joint replacement (11–13). These discrepancies may be caused by a number of variables, including differences in baseline vitamin D status, geographic and ethnic differences, population characteristics, sample size, and so on (14).

Given these inconsistent findings, a better understanding of serum 25(OH)D’s effect on OA is imperative to guide public health recommendations. The purpose of this study is to assess the associations between serum 25(OH)D and osteoarthritis in a large-scale cross-sectional data set from the National Health and Nutrition Examination Survey (NHANES), which represents a nationally representative sample of the US population.

## 2. Materials and methods

### 2.1. Study population

National Health and Nutrition Examination Survey is a continuous national survey conducted by the National Center for Health Statistics, a unit of the Centers for Disease Control and Prevention. In each 2 years cycle, NHANES chose a nationally representative sample of non-institutionalized civilians from the US population using a complicated, multistage probability design. All participants underwent a thorough in-home interview, followed by a detailed physical examination and blood collection in specially equipped mobile examination centers (MECs). The National Center for Health Statistics Research Ethics Review Board approved the NHANES research protocol, which included written, informed consent from all participants following the principles of the Helsinki Declaration. NHANES data have been extensively used to reliably assess the prevalence and relevant risk factors in various chronic illnesses due to the thoroughness of its research methodology. All the NHANES data used in this study were publicly accessible at https://www.cdc.gov/nchs/nhanes/ (accessed date: 15 July 2022).

**FIGURE 1:**
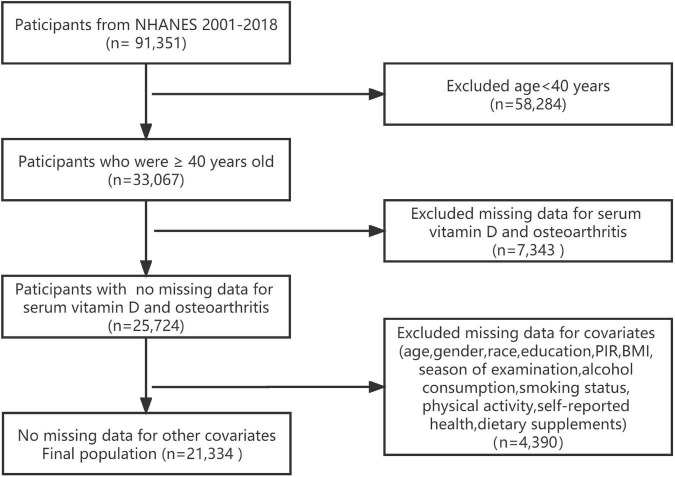
Flowchart of participant selection.

For this analysis, a total of 91,351 participants were enrolled in the NHANES survey over nine cycles (2001–2018). Participants being under 40 years old (*n* = 58,284) were excluded. We further excluded participants with missing data on serum 25(OH)D concentrations (*n* = 3,702), osteoarthritis information (*n* = 3,641), and other covariates (*n* = 4,390). Finally, this study included a large national representative sample (*n* = 21,334) of the general adult US population. The flowchart of the study is shown in Figure 1.

### 2.2. Measurement of serum 25(OH)D

Participants’ blood samples for measuring serum 25(OH)D concentrations were collected at MECs. The samples were promptly frozen at −30°C and shipped to the National Center for Environmental Health (CDC, Atlanta, GA, USA) for analysis.

Total serum 25(OH)D, calculated as the sum of 25(OH)D3 and 25(OH)D2, is the best indicator of vitamin D levels. Serum 25(OH)D concentrations were tested using a radioimmunoassay kit (DiaSorin, Stillwater, MN, USA) in NHANES 2001–2006. The CDC applied a more analytically accurate assay involving ultra-high performance liquid chromatography-tandem mass spectrometry (LC-MS/MS) method in NHANES 2007–2018. To compare 25(OH)D levels across cycles, the CDC standardized 25(OH)D concentrations measured from radioimmunoassays to predicted LC-MS/MS equivalents using regression equations (15). A detailed description of detection methods and standardization can be found on the official website.^1^

### 2.3. Assessment of osteoarthritis

Self-report OA is commonly used for case definition in epidemiological studies (16, 17). Research by March et al. demonstrated 85% consistency between self-reported OA and clinically well-defined OA (17), indicating that OA could typically be reliably self-reported. Each participant was defined as having OA if he/she responded “yes” to the question “Has a doctor or other health professional ever told you that you had arthritis?” and selected “OA” (2001–2010) or “OA or degenerative arthritis” (2011–2018) to the subsequent question “Which type of arthritis was it?”

### 2.4. Covariates

Previous researchers have identified age, gender, race, education level, poverty income ratio (PIR), body mass index (BMI), season of examination, alcohol consumption, smoking status, recreational physical activity, and self-reported health as potential confounders. In this study, confounders were classified in accordance with previous NHANES publications.

Age (years) was used as a continuous variable. Gender was classified as male or female. Race in NHANES was classified as Mexican Americans, Other Hispanics, Non-Hispanic Whites, and Non-Hispanic Blacks, as well as Other Races. In this study, it was collapsed into four categories: Non-Hispanic White, Non-Hispanic Black, Mexican Americans, and Other Race (18). Education was divided into three categories: under high school, high school or equivalent, and college or above (18). Alcohol consumption was defined as the response to the question: “In any one year, had at least 12 drinks of any type of alcoholic beverage?,” and divided into two groups (yes or no). According to the answer to the question: “Have you smoked at least 100 cigarettes in your entire life.” and “Do you now smoke cigarettes?,” smoking status was classified as current smoking, former smoking, and never smoking. Depending on BMI, individuals were classified as low to normal weight (<25 kg/m^2^), overweight (25–30 kg/m^2^), and obesity (≥30 kg/m^2^). The family income was categorized into three groups by the PIR: low income (PIR < 1.3), middle income (1.3 ≤ PIR < 3.5), and high income (PIR ≥ 3.5) (19). There were two examination seasons: November–April (winter) and May–October (summer). The level of recreational physical activity was divided into two categories: active and inactive. Individuals were categorized as active if they reported moderate or vigorous recreational physical activity over the past 30 days (2001–2006) or in a typical week (2007–2018). Those who reported no moderate or vigorous recreational physical activity were categorized as inactive (20). Dietary supplement information was obtained from the questionnaire, which was used to collect detailed information on dietary supplement use. In the current study, dietary vitamin D-containing supplement use was defined as consuming more than one dietary supplement containing vitamin D in the past 30 days.

### 2.5. Statistical analysis

Data management was conducted with R version 4.2.0.^2^ To ensure nationally representative estimates, analyses were adjusted for sampling weights and survey design with the R “survey” package version 4.1-1. The new sample weight was created under the NHANES analytical guidelines. Baseline characteristics were conducted according to different serum 25(OH)D levels (<50 nmol/L; 50–75 nmol/L; 50–75 nmol/L; ≥100 nmol/L) corresponding to the common clinical cut-offs. Continuous variables were summarized using means (standardized error, SE) or medians (interquartile range, 25–75%), and categorical variables were described using proportions (%). Variables were compared using one-way ANOVA (normal distribution), Kruskal–Wallis (skewed distribution) test, and the Chi-square tests (categorical variables), respectively. Multivariate logistic regression analysis was adopted to assess the independent association between serum 25(OH)D and osteoarthritis with different covariates adjusted models. Odds ratio (OR) with a corresponding 95% confidence interval (CI) was calculated using 25(OH)D concentrations both as continuous (per 10 nmol/L increase) and as categorical variables. Tests for linear trend were performed by using the median value of each category as a continuous variable. Subgroup and interaction analysis were performed by multivariate regression analysis adjusting for relevant effect covariates. In addition, sensitivity analyses were performed to assess the robustness of the results. First, considering the potential reverse causation by vitamin D supplement use, we conducted a sensitivity analysis that excluded participants with vitamin D supplement use. Second, as osteoarthritis is more common in older people, participants over 60 years old were re-collected and analyzed. Third, we performed inverse probability of treatment weighting (IPTW) and covariate adjustment using the propensity score (propensity score adjusted regression) to address potential confounders. The following variables were used to generate the models: age, gender, race, education level, PIR, BMI, season of examination, alcohol consumption, smoking status, recreational physical activity, vitamin D supplements, and self-reported health. In all tests, a two-sided *p*-value < 0.05 was considered statistically significant.

## 3. Results

### 3.1. Baseline characteristics of participants

The weighted baseline characteristics stratified by the level of serum 25(OH)D are shown in Table 1. A total of 21,334 participants were included in our study, of whom 48.5% were male, and the mean age was 56.9 years old. The overall prevalence of osteoarthritis was 18.2%. Participants with a higher level of serum 25(OH)D concentrations have a higher prevalence of osteoarthritis than those with a lower level of serum 25(OH)D (<50 nmol: 13.5%, 50–75 nmol: 15.7%, 75–100 nmol: 20.9%, ≥100 nmol/L: 28.6%, *p* < 0.001).

**TABLE 1:** Baseline characteristics of participants aged ≥40 years with or without osteoarthritis according to serum 25(OH)D concentrations in NHANES 2001–2018.

	Serum 25(OH)D concentrations (nmol/L)				
Characteristics	Total (*n* = 21,334)	<50 (*n* = 6,453)	50–75 (*n* = 8,094)	75–100 (*n* = 4,759)	≥100 (*n* = 2,028)
Serum 25(OH)D (nmol/L), mean ± SE	70.7 ± 0.5	37.6 ± 0.2	62.8 ± 0.1	85.6 ± 0.1	122.1 ± 0.8
Age (years), mean ± SE	56.9 ± 0.1	55.4 ± 0.2	55.8 ± 0.2	57.5 ± 0.2	61.2 ± 0.4
**Gender, % (95% CI)**					
Female	51.5 (48.9, 54.1)	53.5 (51.8, 55.2)	46.0 (44.7, 47.3)	50.4 (48.4, 52.4)	67.3 (64.7, 69.9)
Male	48.5 (46.1, 50.9)	46.5 (44.8, 48.2)	54.0 (52.7, 55.3)	49.6 (47.6, 51.6)	32.7 (30.1, 35.3)
**Race/Ethnicity, % (95% CI)**					
Non-Hispanic White	75.4 (70.0, 80.7)	53.7 (50.5, 56.8)	75.9 (73.8, 77.9)	86.3 (84.6, 88.0)	88.8 (87.1, 90.5)
Non-Hispanic Black	9.1 (8.3, 9.9)	23.2 (20.7, 25.7)	6.5 (5.7, 7.3)	3.7 (3.0, 4.4)	3.7 (2.9, 4.5)
Mexican-American	5.8 (4.9, 6.7)	10.2 (8.3, 12.0)	6.6 (5.5, 7.7)	3.0 (2.4, 3.7)	1.4 (0.9, 1.9)
Other	9.8 (8.9, 10.6)	13.0 (11.4, 14.6)	11.0(9.8, 12.2)	7.0(5.9, 8.0)	6.1(4.8, 7.3)
**Season of examination, % (95% CI)**					
Winter	41.2 (37.1, 45.2)	53.6 (48.7, 58.5)	40.1 (35.9, 44.3)	35.2 (31.0, 39.4)	35.1 (29.0, 41.2)
Summer	58.8 (53.1, 64.6)	46.4 (41.5, 51.3)	59.9 (55.7, 64.1)	64.8 (60.6, 69.0)	64.9 (58.8, 71.0)
**Education level, % (95% CI)**					
<High school	14.9 (13.9, 16.0)	20.7 (19.3, 22.1)	16.0 (14.8, 17.3)	11.3 (10.0, 12.7)	9.3(7.5, 11.0)
High school	23.9 (22.3, 25.5)	25.2 (23.5, 27.0)	23.3 (21.8, 24.8)	24.5 (22.8, 26.3)	21.8 (19.2, 24.3)
>High school	61.2 (57.8, 64.5)	54.1 (52.1, 56.0)	60.7 (58.8, 62.6)	64.1 (61.8, 66.4)	69.0 (66.1, 71.9)
**Family income to poverty ratio, % (95% CI)**					
<1.3	16.3 (15.1, 17.5)	24.4 (22.7, 26.1)	16.3 (14.9, 17.7)	12.4 (11.1, 13.7)	10.1(8.5, 11.6)
1.3–3.5	34.3 (32.3, 36.3)	37.4 (35.5, 39.4)	34.6 (33.0, 36.1)	32.6 (30.3, 34.8)	31.8 (28.8, 34.7)
≥3.5	49.4 (46.2, 52.7)	38.2 (35.7, 40.7)	49.2 (46.9, 51.4)	55.1 (52.3, 57.8)	58.2 (54.6, 61.8)
**BMI (kg/m^2^), % (95% CI)**					
<25.0	26.5 (24.9, 28.0)	20.6 (19.2, 21.9)	23.2 (21.9, 24.5)	31.1 (29.2, 33.0)	36.8 (34.2, 39.5)
25.0–30	35.7 (33.7, 37.7)	30.6 (29.0, 32.2)	37.4 (35.9, 38.8)	37.8 (36.2, 39.4)	35.3 (32.8, 37.8)
≥30.0	37.8 (35.9, 39.8)	48.8 (46.9, 50.7)	39.4 (37.7, 41.2)	31.1 (29.1, 33.1)	27.8 (25.6, 30.1)
**Smoking status, % (95% CI)**					
Never smoker	50.9 (48.5, 53.4)	48.1 (46.3, 50.0)	50.9 (49.3, 52.5)	51.4 (49.3, 53.5)	54.9 (52.2, 57.6)
Ever smoker	31.0 (28.9, 33.0)	25.7 (24.2, 27.3)	31.6 (30.1, 33.1)	33.7 (31.9, 35.6)	32.5 (29.9, 35.0)
Current smoker	18.1 (16.9, 19.3)	26.1 (24.6, 27.7)	17.5 (16.3, 18.7)	14.8 (13.3, 16.4)	12.6 (10.6, 14.7)
**Recreational physical activity, % (95% CI)**					
Active	54.8 (51.7, 57.9)	42.2 (40.3, 44.1)	56.1 (54.2, 57.9)	60.6 (58.2, 63.0)	61.0 (58.1, 63.8)
Inactive	45.2 (42.6, 47.8)	57.8 (55.9, 59.7)	43.9 (42.1, 45.8)	39.4 (37.0, 41.8)	39.0 (36.2, 41.9)
**Alcohol consumption (drink/year), % (95% CI)**					
<12	27.9 (26.2, 29.7)	33.9 (32.0, 35.9)	26.1 (24.3, 27.9)	25.3 (23.4, 27.2)	28.6 (25.2, 32.1)
≥12	72.1 (68.0, 76.1)	66.1 (64.1, 68.0)	73.9 (72.1, 75.7)	74.7 (72.8, 76.6)	71.4 (67.9, 74.8)
**Vitamin D supplements, % (95% CI)**					
Yes	46.6 (44.0, 49.2)	20.6 (19.1, 22.1)	42.1 (40.3, 43.8)	59.2 (56.8, 61.6)	79.9 (77.2, 82.6)
No	53.4 (50.7, 56.1)	79.4 (77.9, 80.9)	57.9 (56.2, 59.7)	40.8 (38.4, 43.2)	20.1 (17.4, 22.8)
**Self-reported health, % (95% CI)**					
Fair/poor	18.4 (17.2, 19.5)	26.6 (24.9, 28.3)	17.7 (16.5, 19.0)	14.9 (13.6, 16.2)	13.4 (11.6, 15.2)
Moderate	33.9 (32.2, 35.7)	38.1 (36.6, 39.7)	34.8 (33.4, 36.1)	30.7 (29.0, 32.4)	31.0 (28.0, 34.0)
Excellent/very good	47.7 (44.8, 50.5)	35.2 (33.5, 36.9)	47.5 (45.8, 49.2)	54.4 (52.3, 56.5)	55.7 (52.1, 59.2)
**Osteoarthritis, % (95% CI)**					
Yes	18.2 (16.9, 19.5)	13.5 (12.4, 14.6)	15.7 (14.5, 16.9)	20.9 (19.4, 22.4)	28.6 (25.9, 31.4)
No	81.8 (77.9, 85.6)	86.5 (85.4, 87.6)	84.3 (83.1, 85.5)	79.1 (77.6, 80.6)	71.4 (68.6, 74.1)

Data are summarized as mean ± SE for continuous variables or as proportions (95% CI) for categorical variables. The mean values and percentages are weighted with sampling weights.

The unweighted baseline characteristics of participants with complete data vs. missing data are shown in Supplementary Table 1. Those missing data for serum vitamin D and osteoarthritis were found to be more likely to be older (63.0 vs. 59.3 of those with complete data), non-Hispanic Black (25.7 vs. 19.7% of those with complete data), and female (54.4 vs. 49.5% of those with complete data).

### 3.2. Temporal trends in serum 25(OH)D and osteoarthritis

In this study (Table 2), the mean serum 25(OH)D concentrations significantly increased from 62.1 ± 0.7 nmol/L in 2001–2006 to 76.9 ± 1.0 nmol/L in 2013–2018 (*p* < 0.001). And the prevalence of vitamin D deficiency (<30 nmol/L) reduced from 5.5% (95% CI: 4.5, 6.5%) in 2001–2006 to 3.8% (95% CI: 3.1, 4.4%) in 2013–2018 (*p* < 0.001).

**TABLE 2:** Serum 25(OH)D concentrations and prevalence of serum 25(OH)D concentrations below or above various clinical cut-offs, vitamin D supplement use, osteoarthritis in this study stratified by NHANES survey cycles (2001–2018).

Variables	2001–2006	2007–2012	2013–2018	*p*-Value
Serum 25(OH)D (nmol/L), mean ± SE	62.1 ± 0.7	71.9 ± 0.9	76.9 ± 1.0	<0.001
**25(OH)D cut-off group, % (95% CI)**				
<30 nmol/L	5.5 (4.5, 6.5)	4.8 (3.9, 5.7)	3.8 (3.1, 4.4)	<0.001
<50 nmol/L	28.8 (26.2, 31.3)	21.2 (18.8, 23.5)	17.8 (15.7, 19.9)	<0.001
≥75 nmol/L	25.5 (23.0, 28.1)	42.5 (39.8, 45.2)	48.4 (45.7, 51.1)	<0.001
≥100 nmol/L	4.2 (3.2, 5.2)	13.1 (11.4, 14.9)	18.8 (17.1, 20.5)	<0.001
Vitamin D supplements use, % (95% CI)	45.0 (43.1, 47.0)	45.2 (42.9, 47.5)	49.2 (47.2, 51.2)	0.01
Osteoarthritis, % (95% CI)	14.3 (13.4, 15.2)	16.3 (15.0, 17.7)	23.2 (21.5, 24.8)	<0.001

There was a significant rise in the percentage of persons with high serum 25(OH)D concentrations (≥100 nmol/L) (Table 2) from 4.2% (95% CI: 3.2, 5.2%) in 2001–2006 to 18.8% (95% CI: 17.1, 20.5%) in 2013–2018 (*p* < 0.001), with an obviously increased tendency toward the prevalence of osteoarthritis from 14.3% (95% CI: 13.4, 15.2%) to 23.2% (95% CI: 21.5, 24.8%) (*p* < 0.001).

### 3.3. The association between serum 25(OH)D and osteoarthritis

In this study, multiple regression analysis was employed to assess the association between serum 25(OH)D and osteoarthritis in four models. In model 1, no covariates were adjusted. Model 2 was adjusted for demographics (age, gender, race, education level, PIR, BMI, and season of examination). Model 3 was further adjusted (from model 2) for lifestyle factors (alcohol consumption, smoking status, recreational physical activity, and vitamin D supplement). As there is a close association between vitamin D status and ill health (21), model 4 was further adjusted (from model 3) for self-reported health (Table 3).

**TABLE 3:** Multivariate logistic regression analysis between serum 25(OH)D and osteoarthritis.

Exposure	OR (95% CI)			
	Model 1	Model 2	Model 3	Model 4
Per 10 nmol/L increase	1.11 (1.09, 1.13)^1^	1.08 (1.06, 1.10)^1^	1.07 (1.05, 1.09)^1^	1.07 (1.05, 1.09)^1^
**Clinical cut-offs**				
<50 nmol/L	1 (reference)	1 (reference)	1 (reference)	1 (reference)
50–75 nmol/L	1.19 (1.06, 1.35)^1^	1.23 (1.08, 1.40)^1^	1.22 (1.07, 1.40)^1^	1.25 (1.10, 1.43)^1^
75–100 nmol/L	1.69 (1.48, 1.92)^1^	1.62 (1.41, 1.85)^1^	1.58 (1.38, 1.81)^1^	1.62 (1.42, 1.85)^1^
≥100 nmol/L	2.56 (2.16, 3.04)^1^	1.93 (1.61, 2.32)^1^	1.86 (1.55, 2.25)^1^	1.91 (1.59, 2.30)^1^
*p* for trend	<0.001	<0.001	<0.001	<0.001

OR, odds ratio; 95% CI, 95% confidence intervals.

^1^*p* < 0.001.

Model 1: no covariates were adjusted.

Model 2: adjusted for adjusted for demographics (age, gender, race, education level, PIR, BMI, and season of examination).

Model 3: further adjusted (from model 2) for lifestyle factors (alcohol consumption, smoking status, recreational physical activity, and vitamin D supplements).

Model 4: further adjusted (from model 3) for self-reported health.

We found that a higher level of serum 25(OH)D was associated with a higher risk of osteoarthritis [OR for a 10 nmol/L increase of serum 25(OH)D, model 1, 1.11 (95% CI: 1.09, 1.13); model 2, 1.08 (95% CI: 1.06, 1.10); model 3, 1.07 (95% CI: 1.05, 1.09); model 4, 1.07 (95% CI: 1.05, 1.09); all *p* < 0.001].

Compared with the lowest 25(OH)D group (<50 nmol/L), the fully adjusted OR (model 4) was 1.25 (95% CI: 1.10, 1.43, *p* < 0.001) for the 50–75 nmol/L group, 1.62 (95% CI: 1.42, 1.85, *p* < 0.001) for the 75–100 nmol/L group, and 1.91 (95% CI: 1.59, 2.30, *p* < 0.001) for the ≥100 nmol/L group, indicating a positive relationship between serum 25(OH)D and osteoarthritis with a statistical significance (all *p* < 0.001 for trend).

### 3.4. Sensitivity analyses

After excluding participants with vitamin D supplement use (unweighted *n* = 12,282), serum 25(OH)D was still positively associated with osteoarthritis, either entered as a continuous variable (OR = 1.10; 95% CI: 1.06, 1.13) or as a categorical variable (Table 4).

**TABLE 4:** Sensitivity analyses.

Analysis	Adjusted OR (95% CI)	*p*-Value	*p* for trend
**Excluding participants with vitamin D supplements use^1^**			
Serum 25(OH)D per 10 nmol/L increase	1.10 (1.06, 1.13)	<0.001	
**Clinical cut-offs**			
<50 nmol/L	1 (reference)		<0.001
50–75 nmol/L	1.22 (1.03, 1.45)	0.022	
75–100 nmol/L	1.58 (1.28, 1.95)	<0.001	
≥100 nmol/L	2.07 (1.45, 2.94)	<0.001	
**Participants aged ≥ 60 years^2^**			
Serum 25(OH)D per 10 nmol/L increase	1.07 (1.05, 1.10)	<0.001	
Clinical cut-offs			
<50 nmol/L	1 (reference)		<0.001
50–75 nmol/L	1.23 (1.04, 1.47)	0.020	
75–100 nmol/L	1.59 (1.33, 1.89)	<0.001	
≥100 nmol/L	1.88 (1.50, 2.35)	<0.001	
**Inverse probability treatment weighted analyses^3^**			
<50 nmol/L	1 (reference)		<0.001
50–75 nmol/L	1.24 (1.04, 1.46)	0.014	
75–100 nmol/L	1.66 (1.41, 1.95)	<0.001	
≥100 nmol/L	1.95 (1.57, 2.40)	<0.001	
**Covariate adjustment using the propensity score^3^**			
<50 nmol/L	1 (reference)		<0.001
50–75 nmol/L	1.15 (1.01, 1.32)	0.037	
75–100 nmol/L	1.45 (1.26, 1.68)	<0.001	
≥100 nmol/L	1.68 (1.39, 2.04)	<0.001	

^1^Adjusted for age, gender, race, education level, PIR, BMI, season of examination, alcohol consumption, smoking status, recreational physical activity, and self-reported health.

^2^Adjusted for age, gender, race, education level, PIR, BMI, season of examination, alcohol consumption, smoking status, recreational physical activity, vitamin D supplements, and self-reported health.

^3^The following variables were used to generate the models: age, gender, race, education level, PIR, BMI, season of examination, alcohol consumption, smoking status, recreational physical activity, vitamin D supplements, and self-reported health.

Participants over 60 years old were re-collected and analyzed (unweighted *n* = 10,422). Serum 25(OH)D was still positively associated with osteoarthritis, either entered as a continuous variable (OR = 1.07; 95% CI: 1.05, 1.10) or as a categorical variable (Table 4).

We further used two propensity score methods to address potential confounders. After IPTW and covariate adjustment using the propensity score, the positive association between serum 25(OH)D and osteoarthritis remained (Table 4).

### 3.5. Subgroup analysis

**FIGURE 2:**
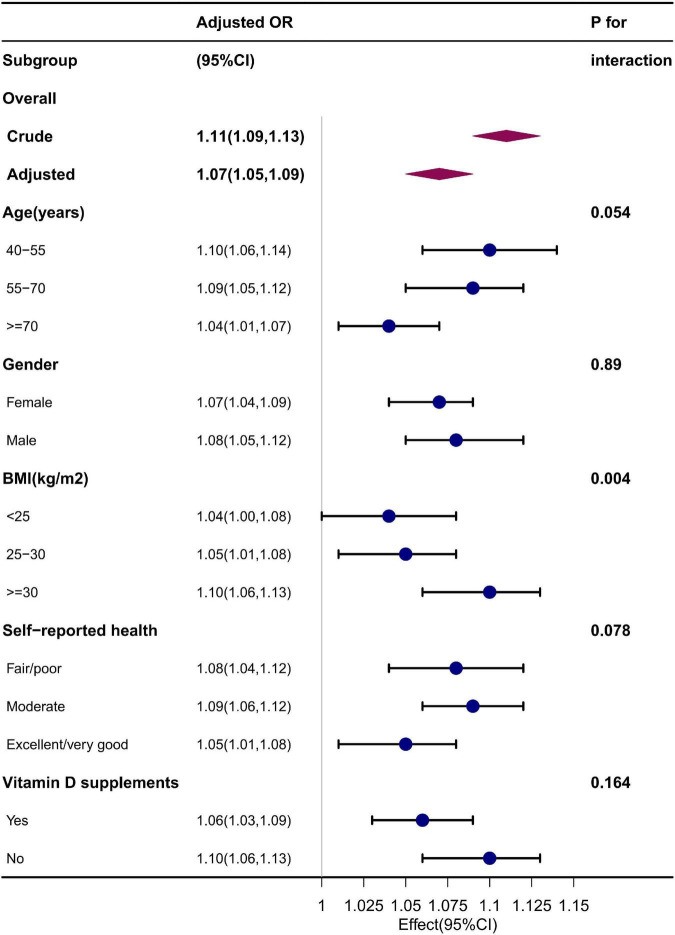
Subgroup analysis. Adjusted for age, gender, race, education level, PIR, BMI, season of examination, alcohol consumption, smoking status, recreational physical activity, vitamin D supplements, and self-reported health.

Subgroup analyses were further performed to explore the association in a different population setting, including age, gender, BMI, self-reported health, and vitamin D supplement use. After adjusting for the confounders, the effect size for each subgroup remains relatively stable, as shown in Figure 2. The interaction test was significant for BMI but not for age, gender, self-reported health, and vitamin D supplement use. The OR for association between serum 25(OH)D and osteoarthritis increased significantly with an increase of BMI (BMI < 25 kg/m^2^, 1.04 [95% CI: 1.00, 1.08]; BMI 25–30 kg/m^2^, 1.05 [95% CI: 1.01, 1.08]; BMI ≥ 30 kg/m^2^, 1.10 [95% CI: 1.06, 1.13]) (*p* = 0.004 for interaction).

## 4. Discussion

In the present cross-sectional survey, we evaluated the association between serum 25(OH)D and the risk of osteoarthritis in a large, nationally representative dataset comprising 21,334 older (>40 years) adults in the United States. Our results showed the higher level of serum 25(OH)D was independently associated with a higher risk of osteoarthritis, which was further demonstrated by subgroup and sensitivity analyses. Meanwhile, we found that BMI might be a modification for the association between serum 25(OH)D and osteoarthritis.

Previous studies on the association between serum 25(OH)D and osteoarthritis did not provide consistent results. These inconsistencies may be partly explained by confounding factors associated with low serum 25(OH)D. For instance, a cross-sectional study of 2,756 participants (1,654 females) found that the presence of osteoarthritis was higher in individuals of the lowest 25(OH)D quartiles group. Yet, individuals in the study were old (mean age 79.04 ± 7.75 years old) and had a much lower level of serum 25(OH)D (23.09 ± 9.15 nmol/L) in females (6). Recent research indicates that low serum 25(OH)D may only be a marker but not a maker of ill-health across many conditions (21). Thus, in older people, vitamin D deficiency may explain as a consequence of the inflammatory process and less sunlight exposure due to low activity levels with osteoarthritis, rather than a cause of disease. In another longitudinal cohort study, the authors found that vitamin D deficiency (<25 nmol/L) predicts incident or worsening of joint pain (7). Regarding the association between vitamin D deficiency and generalized pain (22), the results could not demonstrate vitamin D deficiency independently indicates the incident or worsening of osteoarthritis. Several randomized controlled trials (RCTs) assessing the effect of vitamin D supplement on OA progression did not demonstrate positive outcomes (23). These RCTs are limited by small sample size (<500), short length of supplement (<3 years), patients with severe osteoarthritis and relatively high vitamin D levels (≥50 nmol/L).

Conversely, we found that adults aged 40 years and older with higher serum 25(OH)D status had a higher prevalence of osteoarthritis, which was consistent with several previous researches. A Finland cohort study of 5,274 participants followed up for 10 years (50,134 person-years) showed a significant positive association between the serum 25(OH)D and the risk of knee and hip osteoarthritis (13). In an Australian study of 9,135 individuals followed up for 9.1 (SD 2.7) years, they found that higher serum 25(OH)D was associated with an increment of hip replacement due to osteoarthritis (12). In another independent cohort followed up for 15.7 (SD 4.6) years, they found similar results (11).

Because vitamin D is believed to have beneficial effects on bone, muscle, and inflammation, it may be easier to illustrate the association between serum 25(OH)D deficiency and the risk of osteoarthritis. But we should consider the problem from different angles or in combination. The bone-cartilage unit is a functional complex formed by subchondral bone and cartilage that play a vital role in the pathogenesis of osteoarthritis at both mechanical and biochemical levels (24, 25). In the course of the disease, the subchondral bone is progressively remodeled and becomes thicker and stiffer, resulting in a less deformable subchondral bone with impaired shock absorbing capacity (24, 25). As a result, excessive load is directly transferred to the articular cartilage, resulting in cartilage loss and radiographic osteoarthritis (26). Vitamin D plays a vital role in calcium absorption and bone metabolism, and it is correlated with increased bone mineral density (BMD) (4). Furthermore, osteoblasts from OA patients exhibit a higher vitamin D-induced bone-forming capacity (5). As a consequence, vitamin D’s effect on bone formation might result in subchondral bone sclerosis and osteophyte, and there is growing evidence that vitamin D is related to tibial subchondral BMD (27). Consistent with these findings, some studies have indicated that higher BMD could contribute to osteoarthritis onset and progression (28). We found that obesity acts as a modifier of the association between serum 25(OH)D and osteoarthritis, as the odds ratio in the obesity group is much higher than in the non-obesity group. The mechanism for this interaction may be explicable. In weight-bearing joints, such as the knee, subchondral bone remodels quickly in response to mechanical loading (25). Therefore, higher BMI combined with vitamin D may drastically accelerate the subchondral sclerosis progress. Further studies to elucidate this association at biochemical and mechanical levels may help develop new ways to treat and prevent OA.

Over the past few decades, a wealth of studies have connected vitamin D deficiency with variety non-skeletal diseases, which has generated strong interest and even excessive enthusiasm about the promising benefits of vitamin D supplement. The serum levels of 25(OH)D to be considered sufficiency varied from 25–30 to 50, 75, and even >100 nmol/L (29). The average serum 25(OH)D in this study was as high as 70.72 ± 0.54 nmol/L, with an increasing tendency. Hence, recognizing the threshold effect of serum 25(OH)D on osteoarthritis is critical. However, in recent years, many RCTs revealed that vitamin D supplement for vitamin D-replete individuals (>50 nmol/L) do not provide demonstrable health benefits (30). There is increasing evidence that high doses of vitamin D supplement, or high serum 25(OH)D poses risks besides just hypercalcemia or hypercalciuria. Some high-quality studies found that high-dose vitamin D supplement increased the risk of fractures and falls (31, 32), and these regimens are related with serum 25(OH)D higher than 100–112 nmol/L (40–45 ng/ml) (33). In this study, there was a dramatic rise in the percentage of participants with high serum 25(OH)D, and as high as 18.77% of participants achieved ≥100 nmol/L in NHANES 2013–2018. We also found that the risk of osteoarthritis corresponded in time with an increase in serum 25(OH)D. Meanwhile, there were only 3.75–5.49% of participants with severe vitamin D deficiency (<30 nmol/L). So, we should be more concerned about vitamin D overdose in the future, and more high-quality clinical trials with various baseline serum 25(OH)D are required to further distinguish the threshold effect of serum 25(OH)D on osteoarthritis. The availability of new data, however, vitamin D supplement for vitamin D-replete (≥50 nmol/L) individuals with OA risk factors (especially for obesity) may be unwise.

The strengths of the current study include a large sample size, the availability of comprehensive information for adjusting a multitude of potential confounders and the use of a nationally representative survey of U.S. civilian, which facilitates the generalization of our findings. Our study also has several limitations. First, serum 25(OH)D levels are known to be influenced by many different conditions. Although, we considered and adjusted many potential confounders in the study, residual confounders may still exist. And the assessment of serum 25(OH)D with a single measurement has the potential to misclassify the individuals’ long-term 25(OH)D status. However, several sensitivity analyses were conducted to demonstrate the reliability of the findings, and serum 25(OH)D is relatively stable over time (34). Second, there are some differences between the baseline characteristics of respondents and non-respondents, which raise the possibility of non-response bias. Third, we have no information on OA sites and related radiographic imaging. Therefore, we cannot analyze data stratified by OA sites and severity. Although some individuals with early-stage asymptomatic osteoarthritis may have been missed, it is unlikely to significantly change the findings. Finally, the most concern is that osteoarthritis is prevalent among the elderly who are more aware of vitamin D supplement, the higher serum vitamin D in OA subjects may explain as a consequence. However, indications for vitamin D supplement might mainly be older or bone health rather than osteoarthritis. In addition, the NHANES data have been extensively utilized to reliably evaluate the risk factors or prevalence of many chronic diseases like OA. For instance, poor serum 25(OH)D status associated with risk of T2DM was documented in some NHANES studies, in line with other large long-term prospective studies (4). To reduce the possibility of reverse causation, we excluded people with vitamin D supplement use in the sensitivity analysis. Nevertheless, given its cross-sectional design, reverse causation cannot be entirely removed, although this seems less likely.

## 5. Conclusion

In summary, this represents the largest study to demonstrate an association between serum 25(OH)D and the risk of osteoarthritis. We found a significantly positive association and, for the first time, reported that the association was modified by BMI. This study raises concerns about the potential adverse effects of high serum 25(OH)D on osteoarthritis, particularly among obese individuals. More well-designed studies are still needed to validate our findings in future.

## Data availability statement

The datasets presented in this study can be found in online repositories. The names of the repository/repositories and accession number(s) can be found in the article/Supplementary material.

## Ethics statement

The studies involving human participants were reviewed and approved by **t**he National Center for Health Statistics Research Ethics Review Board. The patients/participants provided their written informed consent to participate in this study.

## Author contributions

JX and JL designed and conceived the research, supervised the study, and revised drafts of the manuscript. GY directed the analytic strategy. GY, YL, and HD analyzed the data and interpreted the results. GY and YL drafted the manuscript. All authors read and agreed to the published version of the manuscript.

## Footnotes

1
https://www.cdc.gov/nchs/nhanes/vitamind/analyticalnote.aspx

2
http://www.R-project.org
