# Supplementary material for: Association between serum 25-hydroxyvitamin D and osteoarthritis: A national population-based analysis of NHANES 2001–2018
Source: Front Nutr. 2023 Feb 28;10:1016809. doi: 10.3389/fnut.2023.1016809 (PMC10011108; doi:10.3389/fnut.2023.1016809)
Supplement: Supplementary file 1 [file Table_1.DOC]

**Table S1**. Baseline characteristics of participants with complete data vs missing data for serum vitamin D and osteoarthritis

| **Characteristics** | **Complete data** | **Missing data for serum vitamin D and osteoarthritis** |
| --- | --- | --- |
|  | **Unweighted n=21334** | **Unweighted n=7343** |
| **Age(years), Mean ± SD** | 59.3 ± 12.5 | 63.0 ± 12.8 |
| **Gender, %** |  |  |
| Female | 10555 (49.5) | 3998 (54.4) |
| Male | 10779 (50.5) | 3345 (45.6) |
| **Race/ethnicity, %** |  |  |
| Non-Hispanic White | 10503 (49.2) | 3312 (45.1) |
| Non-Hispanic Black | 4199 (19.7) | 1884 (25.7) |
| Mexican-American | 3232 (15.1) | 986 (13.4) |
| Other | 3400 (15.9) | 1161 (15.8) |
| **Season of examination, %** |  |  |
| Winter | 9963 (46.7) | 2709 (46.7) |
| Summer | 11371 (53.3) | 3095 (53.3) |
| **Education level, %** |  |  |
| <High school | 5436 (25.5) | 2642 (36.2) |
| High school | 4906 (23) | 1798 (24.7) |
| >High school | 10992 (51.5) | 2854 (39.1) |
| **Family income to poverty ratio, %** |  |  |
| <1.3 | 5705 (26.7) | 2115 (33.8) |
| 1.3-3.5 | 8150 (38.2) | 2454 (39.2) |
| ≥3.5 | 7479 (35.1) | 1695 (27.1) |
| **BMI (kg/m²), %** |  |  |
| <25.0 | 5470 (25.6) | 1384 (25) |
| 25.0-30 | 7702 (36.1) | 1831 (33) |
| ≥30.0 | 8162 (38.3) | 2330 (42) |
| **Smoking status, %** |  |  |
| Never smoker | 10719 (50.2) | 3560 (48.6) |
| Ever smoker | 6637 (31.1) | 2383 (32.5) |
| Current smoker | 3978 (18.6) | 1380 (18.8) |
| **Recreational physical activity, %** |  |  |
| Active | 10210 (47.9) | 2632 (35.9) |
| Inactive | 11124 (52.1) | 4700 (64.1) |
| **Alcohol consumption (drink/year), %** |  |  |
| <12 | 7094 (33.3) | 1874 (38) |
| ≥12 | 14240 (66.7) | 3060 (62) |
| **Vitamin D supplements, %** |  |  |
| Yes | 9052 (42.4) | 2914 (39.8) |
| No | 12282 (57.6) | 4399 (60.2) |
| **Self-reported health, %** |  |  |
| Fair/poor | 5495 (25.8) | 2763 (37.7) |
| Moderate | 7672 (36) | 2503 (34.1) |
| Excellent/very good | 8167 (38.3) | 2066 (28.2) |
